# Supplementary material for: Implicit HbA1c Achieving 87% Accuracy within 90 Days in Non-Invasive Fasting Blood Glucose Measurements Using Photoplethysmography
Source: Bioengineering (Basel). 2023 Oct 16;10(10):1207. doi: 10.3390/bioengineering10101207 (PMC10604272; doi:10.3390/bioengineering10101207)
Supplement: Supplementary file 1 [file bioengineering-10-01207-s001.zip › bioengineering-2623404-supplementary.pdf]

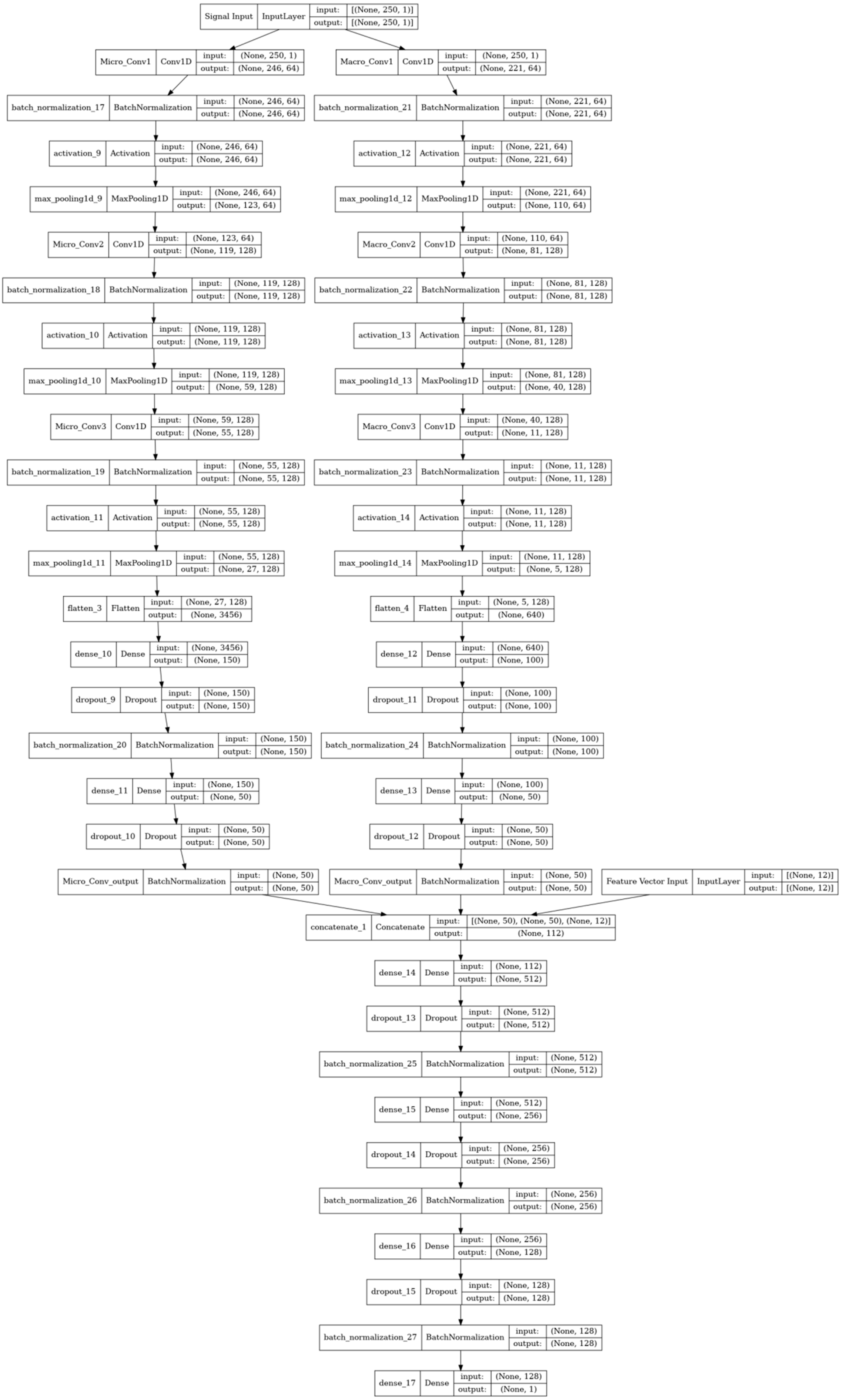
Signal Input | InputLayer | input: [(None, 250, 1)] | output: [(None, 250, 1)]
Micro_Conv1 | Conv1D | input: (None, 250, 1) | output: (None, 246, 64)
Macro_Conv1 | Conv1D | input: (None, 250, 1) | output: (None, 221, 64)
batch_normalization_17 | BatchNormalization | input: (None, 246, 64) | output: (None, 246, 64)
batch_normalization_21 | BatchNormalization | input: (None, 221, 64) | output: (None, 221, 64)
activation_9 | Activation | input: (None, 246, 64) | output: (None, 246, 64)
activation_12 | Activation | input: (None, 221, 64) | output: (None, 221, 64)
max_pooling1d_9 | MaxPooling1D | input: (None, 246, 64) | output: (None, 123, 64)
max_pooling1d_12 | MaxPooling1D | input: (None, 221, 64) | output: (None, 110, 64)
Micro_Conv2 | Conv1D | input: (None, 123, 64) | output: (None, 119, 128)
Macro_Conv2 | Conv1D | input: (None, 110, 64) | output: (None, 81, 128)
batch_normalization_18 | BatchNormalization | input: (None, 119, 128) | output: (None, 119, 128)
batch_normalization_22 | BatchNormalization | input: (None, 81, 128) | output: (None, 81, 128)
activation_10 | Activation | input: (None, 119, 128) | output: (None, 119, 128)
activation_13 | Activation | input: (None, 81, 128) | output: (None, 81, 128)
max_pooling1d_10 | MaxPooling1D | input: (None, 119, 128) | output: (None, 59, 128)
max_pooling1d_13 | MaxPooling1D | input: (None, 81, 128) | output: (None, 40, 128)
Micro_Conv3 | Conv1D | input: (None, 59, 128) | output: (None, 55, 128)
Macro_Conv3 | Conv1D | input: (None, 40, 128) | output: (None, 11, 128)
batch_normalization_19 | BatchNormalization | input: (None, 55, 128) | output: (None, 55, 128)
batch_normalization_23 | BatchNormalization | input: (None, 11, 128) | output: (None, 11, 128)
activation_11 | Activation | input: (None, 55, 128) | output: (None, 55, 128)
activation_14 | Activation | input: (None, 11, 128) | output: (None, 11, 128)
max_pooling1d_11 | MaxPooling1D | input: (None, 55, 128) | output: (None, 27, 128)
max_pooling1d_14 | MaxPooling1D | input: (None, 11, 128) | output: (None, 5, 128)
flatten_3 | Flatten | input: (None, 27, 128) | output: (None, 3456)
flatten_4 | Flatten | input: (None, 5, 128) | output: (None, 640)
dense_10 | Dense | input: (None, 3456) | output: (None, 150)
dense_12 | Dense | input: (None, 640) | output: (None, 100)
dropout_9 | Dropout | input: (None, 150) | output: (None, 150)
dropout_11 | Dropout | input: (None, 100) | output: (None, 100)
batch_normalization_20 | BatchNormalization | input: (None, 150) | output: (None, 150)
batch_normalization_24 | BatchNormalization | input: (None, 100) | output: (None, 100)
dense_11 | Dense | input: (None, 150) | output: (None, 50)
dense_13 | Dense | input: (None, 100) | output: (None, 50)
dropout_10 | Dropout | input: (None, 50) | output: (None, 50)
dropout_12 | Dropout | input: (None, 50) | output: (None, 50)
Micro_Conv_output | BatchNormalization | input: (None, 50) | output: (None, 50)
Macro_Conv_output | BatchNormalization | input: (None, 50) | output: (None, 50)
Feature Vector Input | InputLayer | input: [(None, 12)] | output: [(None, 12)]
concatenate_1 | Concatenate | input: [(None, 50), (None, 50), (None, 12)] | output: (None, 112)
dense_14 | Dense | input: (None, 112) | output: (None, 512)
dropout_13 | Dropout | input: (None, 512) | output: (None, 512)
batch_normalization_25 | BatchNormalization | input: (None, 512) | output: (None, 512)
dense_15 | Dense | input: (None, 512) | output: (None, 256)
dropout_14 | Dropout | input: (None, 256) | output: (None, 256)
batch_normalization_26 | BatchNormalization | input: (None, 256) | output: (None, 256)
dense_16 | Dense | input: (None, 256) | output: (None, 128)
dropout_15 | Dropout | input: (None, 128) | output: (None, 128)
batch_normalization_27 | BatchNormalization | input: (None, 128) | output: (None, 128)
dense_17 | Dense | input: (None, 128) | output: (None, 1)

Supplementary Figure S1. Detailed neural network structure of our deep learning model. The model uses two input layer for signal vector and feature vector. The signal vector takes in the segmented PPG waveform data while feature vector takes in the HbA1c value and other PPG extracted features.
